# Did the organization of primary care practices during the COVID-19 pandemic influence quality and safety? – an international survey

**DOI:** 10.1186/s12913-024-11173-y

**Published:** 2024-06-14

**Authors:** Mats Eriksson, Karin Blomberg, Eva Arvidsson, Esther Van Poel, Sara Ares-Blanco, Maria Pilar Astier-Peña, Claire Collins, Jonila Gabrani, Neophytos Stylianou, Victoria Tkachenko, Sara Willems

**Affiliations:** 1 Faculty of Medicine and Health, School of Health Sciences, Örebro University, Örebro, Sweden; 2 Futurum, Region Jönköping County, Jönköping, Sweden; 3 Department of Health, Medicine and Caring Sciences, Linköping University, Linköping, Sweden; 4 Department of Public Health and Primary Care, Ghent University, Ghent, Belgium; 5 Federica Montseny Health Centre, Gerencia Asistencial de Atención Primaria, Servicio Madrileño de Salud, Madrid, Spain; 6 Healthcare Quality Territorial Unit, Territorial Health Directorate, Institute of Health of Catalonia, Camp de Tarragona, Barcelona, Spain; 7 Irish College of General Practitioners, Dublin, Ireland; 8 University of Medicine, Tirana, Albania; 9 Department of data analysis, NS Intelligence Solutions Ltd, Nicosia, Cyprus; 10 Akesis Home Care, Nicosia, Cyprus; 11 Department of Family Medicine, Shupyk National Healthcare University of Ukraine, Kyiv, Ukraine

**Keywords:** COVID-19, International comparison, Interprofessional collaboration, Multiprofessional, Infection prevention and control, Pricov-19, Quality of care

## Abstract

**Background:**

Changes in demographics with an older population, the illness panorama with increasing prevalence of non-communicable diseases, and the shift from hospital care to home-based care place demand on primary health care, which requires multiprofessional collaboration and team-based organization of work. The COVID-19 pandemic affected health care in various ways, such as heightened infection control measures, changing work practices, and increased workload.

**Objectives:**

This study aimed to investigate the association between primary care practices’ organization, and quality and safety changes during the COVID-19 pandemic.

**Design:**

Data were collected from 38 countries in a large online survey, the PRICOV-19 study. For this paper, the participating practices were categorized as “Only GPs”, comprising practices with solely general practitioners (GPs) and/or GP trainees, without any other health care professionals (*n* = 1,544), and “Multiprofessional,” comprising practices with at least one GP or GP trainee and one or more other health professionals (*n* = 3,936).

**Results:**

Both categories of practices improved in infection control routines when compared before and during the COVID-19 pandemic. A larger proportion of the multiprofessional practices changed their routines to protect vulnerable patients. Telephone triage was used in more “Multiprofessional” practices, whereas “Only GPs” were more likely to perform video consultations as an alternative to physical visits. Both types of practices reported that the time to review new guidelines and scientific literature decreased during the pandemic. However, both had more meetings to discuss directives than before the pandemic.

**Conclusions:**

Multiprofessional teams were keener to introduce changes to the care organization to protect vulnerable patients. However, practices with only GPs were found to be more aligned with video consultations, perhaps reflecting the close patient-doctor relationship. In contrast, telephone triage was used more in multiprofessional teams.

## Background

Providing high-quality primary health care (PHC) is essential to address the challenges of achieving successful universal health coverage today [1]. A worldwide aging population, rising numbers of individuals living with various chronic illnesses and/or experiencing adverse effects from different medical treatments, and the shift from hospital care to home-based care are all placing demands on primary care. The population’s diverse and complex needs require the involvement of healthcare professionals with various competencies, as no single discipline can provide comprehensive care [2]. Consequently, ensuring sustainable primary health care necessitates a well-established organization and collaboration both internally among professionals and externally across various levels of care [1, 3]. However, the composition of teams may vary across general practices [4, 5]. These teams range from small practices with only one general practitioner (GP) to larger teams with many doctors, nurses, secretarial staff, and additional professionals [6]. According to the World Health Organization [1], primary health care is defined as a “whole-of-society approach to health that aims to maximize the level and distribution of health and well-being through three components: (a) primary care and essential public health functions as the core of integrated health services; (b) multisectoral policy and action; and (c) empowered people and communities.” Given this definition’s broad scope, multiple professions must work together effectively. A multiprofessional team is best suited to provide continuous, comprehensive, coordinated, and people-centered care [1]. Physicians often lead these teams and may include generalist medical practitioners (such as family doctors and general practitioners), physician assistants, nurses, specialist nurses, community health workers, pharmacists, social workers, dietitians, mental health counsellors, psychologists, physiotherapists, occupational therapists, patient educators, dentists, podiatrists, midwives, as well as managers and support staff [1, 4].

### Variations in organization

In Europe, the mix of professions that make up the primary care workforce may differ from country to country, but GPs or family medicine practitioners are often considered the core of primary care. The skills and competencies employed by the workforce also vary significantly across countries, as does the training, system-level funding and facilities, thus demonstrating the heterogeneity of primary health care across different countries in Europe [2, 7].

In countries such as France and Germany, where many GPs are self-employed professionals working in single practices for around 50 h weekly and are remunerated mainly according to the services provided, gatekeeping has become almost antithetic. In Spain, this role has been switched from physicians to multifunctional facilities, which are planned to guarantee high access to their catchment populations. Consistently, GPs have become full-time employees of the Spanish national health service (NHS), working in multiprofessional teams to limit redundant procedures in secondary care. In Sweden GPs primary care is provided by both public and private practices. GPs work in teams with other health professionals where nurses have an important function in telephone triage. The role of gatekeeping is expected from secondary care but difficult to live up to because PHC is undersized compared to hospitals. In Italy, GPs are still mainly single-handed and isolated professionals paid per capita, who work in their own practices and offer limited access to their patients. Consequently, GPs play a scant gatekeeping role in the Italian NHS, as highlighted by the recent pandemic. In Germany, outpatient consultants in internal medicine play a very similar role to GPs in providing primary care, stressing that both specialists are generalists by definition [2].

### Teamwork and task shifting

The effect of teamwork in the context of primary care has been studied and, for example,

fewer diabetes-specific ambulatory care sensitive conditions and better glycaemic control have been found for practices that work in teams [8], while other studies found a mix of positive and negative correlations [9] or no relationship between teamwork and quality of care [10]. According to a study examining quality in primary care, various connections among various professions were integral. The study revealed that the number of GPs positively correlated with both GPs’ perceived job satisfaction and patients’ satisfaction. Conversely, the number of nurses and other healthcare professionals was found to have an inverse relationship with these outcomes. Gibson et al. conclude that different healthcare professionals are not interchangeable. Therefore, the outcomes in primary care are contingent upon the team’s composition [11].

Working collaboratively across multiple professions is necessary when resources are limited. The growing number of patients with chronic conditions, higher demands from patients, and shortages in health care professionals, especially GPs and nurses, are increasing problems in many countries [3]. The lack of GPs has been addressed by improving conditions for recruiting and retaining of primary care physicians [12], but this is often insufficient. Then, primary care capacity can also be increased by task-shifting, i.e. letting nurses, nurse practitioners, physician assistants and pharmacists take over tasks traditionally performed by a GP. Also, non-licensed healthcare professionals, can address preventive and chronic care needs by functioning as health coaches and supporting patient self-care using new technology [13]. The degree of task-shifting also varies among countries [14]. Maier et al. found in a study from 2016 that out of 39 OECD countries, 28% showed extensive task-shifting, 41% limited task-shifting, and the rest, 31%, no or almost no task-shifting [15]. Moreover, just as with task-shifting, changing traditions and finding new work methods is not without problems. There may be boundaries between areas of responsibility for different professional groups that are difficult to change, both by tradition and attitudes as well as by regulation [14]. Nevertheless, in Spain, where most practices are multiprofessional teams, task-shifting to nurses increased during COVID-19 pandemic [16] and in Ireland, the perception of GPs regarding pharmacists working in PHC is positive [17].

A Cochrane review suggests that professional substitution, specifically the provision of care by nurses compared to care provided by general practitioners (GPs), yields equivalent or superior health outcomes across various patient conditions [18]. For example, blood pressure outcomes and patients’ satisfaction in nurse-led primary care tend to improve. However, nurses tend to have longer consultations and more numbers of return visits than GPs. It has been concluded that potential cost savings from professional substitution depend on the healthcare professionals’ salaries involved. Still, this conclusion should be viewed with caution given that only one study was powered to assess the equivalence of care, several included studies had methodological limitations, and patient follow-up was generally 12 months or less [18]. Another Cochrane review from 2019 [19] concluded that patients, doctors, and nurses can accept nurse-doctor substitution, but it depends on the type of service. There are also potential difficulties. Nurses who take on new tasks want respect and collaboration from doctors and training and supervision, which is not always met [20]. Task-shifting may also cause difficulties in collaborating and accepting to hand over, or takeover of tasks [19]. Van Schalkwyk et al. present a framework for task-shifting and points out that with adequate planning, resources, education, training, and transparency task-shifting can contribute to health systems strengthening [21].

### Quality and safety in primary health care

There have been several attempts to define quality in health care [22, 23], looking at e.g. structure indicators, process indicators and clinical outcomes [23]. In this project we have investigated structural indicators, meaning how the PHC practices have organized their work to meet the needs of their patients, and how this have changed during the pandemic. Safety issues is one part of quality, in this case meaning how the staff and other patients are protected from infectious diseases, but also how groups with special needs were guaranteed care according to their situation, despite the impact of the pandemic.

Teamwork and partially taking over each other’s tasks require education, training and new attitudes [3, 4]. According to Akman et al., more evidence is needed on the association between multiprofessional practices and cost, quality, and health outcomes [4]. This is even more the case in the context of the COVID-19 pandemic. Recent studies show that it was difficult to continue with teamwork as usual during the COVID-19 pandemic due to changed working methods such as physical distancing and a shift towards telemedicine [24–26]. In the midst of the COVID-19 pandemic, primary care was faced with new demands and responsibilities while also dealing with a shortage of healthcare professionals, leading to an increased workload and burden on the staff [27, 28]. It remains unclear whether practices with a multiprofessional workforce were better equipped to adapt to these changes, or if smaller practices with fewer different professionals, or even solely physicians, had an easier time coping with the challenges posed by the pandemic.

## Objectives

This study aimed to investigate the correlation between primary care practices’ (PCPs) organizational structure (multiprofessional vs. solely GPs), and important aspects of their quality and safety functioning during the COVID-19 pandemic. Specifically, the study sought to examine the correlation between the workforce composition of PCPs and their working routines, outreach efforts undertaken to reach vulnerable populations, and the utilization of guidelines and recommendations in practice.

## Methods

### Study design and setting

In the summer of 2020, an international consortium of more than 45 research institutes was formed under the coordination of the ‘Equity in Health care’ research unit at Ghent University (Belgium) to set up the PRICOV-19 study. This multi-country cross-sectional study aimed to research how PCPs were organized during the COVID-19 pandemic to guarantee high-quality care; how the task roles changed, and the pandemic impacted the well-being of care providers and whether differences could be found between types of PCPs and/or healthcare systems. Data were collected in 37 European countries and Israel [29].

### Data collection

Data were collected by means of an online self-reported questionnaire among PCPs. The questionnaire was developed at Ghent University in multiple phases, including a pilot study among 159 PCPs in Flanders (Belgium). The questionnaire consisted of 53 items divided into six topics: (a) infection prevention; (b) patient flow for COVID- and non-COVID care; (c) dealing with new knowledge and protocols; (d) communication with patients; (e) collaboration and well-being of the respondent; and (f) characteristics of the respondent and practice. The questionnaire was translated into 38 languages following a standard procedure and the English language version can be found in the protocol paper [29].

The Research Electronic Data Capture (REDCap) platform was used to host the questionnaire in all languages, send out invitations to the national samples of PCPs and securely store the answers from the participants [30].

Data were collected between November 2020 and December 2021, except for Belgium, where data were partially collected earlier. Data collection varied in duration between countries from 3 to 35 weeks. The consortium partner(s) recruited PCPs in each partner country following a pre-defined recruitment procedure. Drawing a randomized sample among all PCPs in the country was preferred over convenience sampling, whereas some countries contacted all PCPs or had to use a convenience sample. Partners logged all the steps taken in the sampling procedure. PRICOV-19 aimed to sample between 80 and 200 PC practices per country, depending on the national number of PCPs. However, since there was no funding for this study and coordinators recruited practices voluntarily, enforcing a specific recruitment strategy or response rates was impossible. Per PCP, one questionnaire was completed, preferably by a GP or a staff member familiar with the practice organization. The overall response rate was 22.0% ranging from 1.6% in Denmark to 94.3% in Bulgaria [31].

### Research ethics

The study was conducted according to the guidelines of the Declaration of Helsinki. The Research Ethics Committee of Ghent University Hospital approved the protocol of the PRICOV-19 study (BC-07617). Research Ethics Committees in the different partner countries gave additional approval if needed. All participants gave informed consent on the first page of the online questionnaire. All data was anonymized, and all raw data that could lead to the identification of the participants was permanently removed.

### Data analysis

Ghent University was responsible for the data cleaning of the international data and the database version 8 was used for this analysis, consisting of the cleaned data of 38 countries [29]. The survey was completed by 8,958 PCPs, of which 5,484 provided data on the number of GPs and GP trainees working at their practice, making them eligible for inclusion in this paper’s analysis.

The participating PCPs were categorized into two groups: “Only GPs,” comprising practices with solely GPs and/or GP trainees, without any other professionals (*n* = 1,544), and “Multiprofessional,” comprising practices with at least one GP or GP trainee and one or more other professionals (*n* = 3,936). The professional disciplines that were included were practice manager, dietician or nutritionist, health promotor, physiotherapist or manual therapist or osteopath, social worker, podologist, psychologist, and nurse or nurse assistant. Receptionists and cleaning employees were not included in defining the multiprofessional nature of the practice.

The two categories were compared on how their working routines regarding quality and safety tasks as infection control changed, adherence to and opinions on guidelines and recommendations, and outreach to vulnerable groups.

The data is presented with medians and quartiles 1 and 3 (q1-q3), or proportions (%). Differences between categories were analysed with chi-square test or Mann-Whitney U-test, and the significance level was set to *p* < 0.05. SPSS (IBM SPSS Statistics for Macintosh, Version 28.0. Armonk, NY: IBM Corp) was used for the analyses.

## Results

**Fig. 1:**
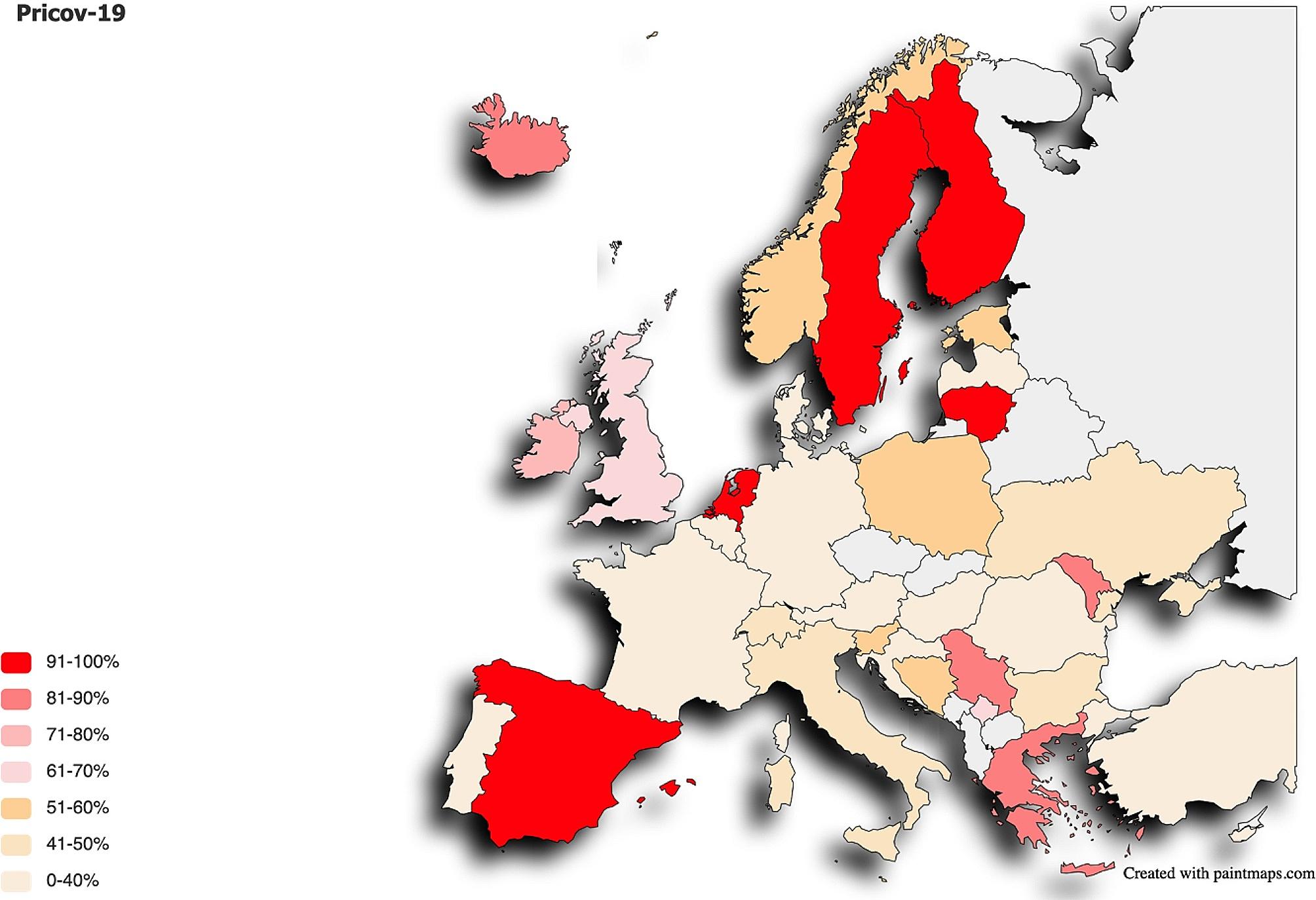
Reported proportion of multiprofessional practices in the participating countries

This paper is built on answers from 5,484 PCPs in Austria, Belgium, Bosnia and Herzegovina, Bulgaria, Cyprus, Croatia, Czech Republic, Denmark, Estonia, Finland, France, Germany, Greece, Hungary, Iceland, Ireland, Italy, Israel, Kosovo, Latvia, Lithuania, Luxembourg, Malta, Moldavia, North Macedonia, Norway, Poland, Portugal, Romania, Serbia, Slovenia, Spain, Sweden, Switzerland, The Netherlands, Turkey, United Kingdom, and Ukraine. The map in Fig. 1 shows the proportion of multiprofessional practices in the participating countries. The “Only GPs” practices had a median of 1 GP working at their practice (q1-q3: 1–3), whereas the corresponding figure for the “Multiprofessional” practices was 3 (q1-q3: 1–6).

### Working routines and infection control

The survey investigated changes in infection control protocols between pre-pandemic and pandemic periods. Before the pandemic, a higher proportion of “Only GPs” practices did not use a detailed cleaning protocol compared to “Multiprofessional” practices. This disparity persisted during the pandemic, although “Only GPs” practices improved more than “Multiprofessional” practices. Additionally, “Only GPs” practices were more likely to provide hand sanitizer for home visits, while “Multiprofessional” practices were better at offering hand sanitizer at the door or in the waiting room and using separate medical bags for home visits to patients suspected of having an infection.

Both practice types showed improvement across all of these parameters (Table 1).

**Table 1:** Changes in infection control routines from before to during COVID-19 pandemic. Significant differences in bold

	Before the pandemic			During the pandemic			Change		
“**How often did it occur that …”**	**Only GPs**	**Multipro-fessional**	*p***-value**	**Only GPs**	**Multipro-fessional**	*p***-value**	**Only GPs**	**Multipro-fessional**	*p***-value**
Detailed cleaning protocol never was used	26.7%	13.6%	**< 0.001**	16.6%	8.3%	**< 0.001**	10.1	5.3	**< 0.001**
Hand sanitizer never was provided in every room	12.5%	11.6%	0.131	5.3%	5.5%	0.101	7.2	6.1	0.142
Hand sanitizer never was provided for home visits	17.3%	18.6%	**0.006**	5.9%	7.1%	**< 0.001**	11.4	11.5	0.942
Hand sanitizer never provided at door or in waiting room	54.2%	46.4%	**< 0.001**	8.4%	6.9%	0.159	45.8	39.5	0.738
Separate medical bag never used for home visit to suspected infectious patients	68.5%	60.2%	**< 0.001**	43.2%	33.8%	**< 0.001**	25.3	26.4	0.792

Most participating PCPs had implemented routine measures to safeguard their staff from infection. These measures included triage, discontinuing waiting room use, and making structural modifications to the reception area. Such measures were more prevalent in the “Multiprofessional” practices. These practices also utilized telephone triage to determine the need for type and urgency of visit more frequently, while “Only GPs” practices were more likely to conduct video consultations (Table 2).

**Table 2:** Safeguarding the staff during the COVID-19 pandemic. Significant differences in bold

How is this practice safeguarding the well-being of the staff since the COVID-19 pandemic?	Only GPs	Multi-professional	*p*-value
Performing triage before patient entering	65.4%	77.2%	**< 0.001**
Performing telephone triage	77.4%	79.8%	**0.038**
Limiting the number of patients in the waiting room	86.7%	85.4%	0.268
No longer using the waiting room	10.0%	14.4%	**< 0.001**
Increasing infection control practices	75.9%	77.9%	0.068
Making structural changes to the reception area	45.6%	53.8%	**< 0.001**
Performing video consultations	43.2%	38.2%	**< 0.001**
Changing repeat prescription approach	61.9%	62.3%	0.420
Using e-script or healthmail for prescriptions	65.2%	75.0%	**< 0.001**

To explore whether there were any changes in the patient flow during the pandemic and how it impacted accessibility, the survey presented a series of indicative scenarios to the respondents. The “Only GPs practices” reported better management of urgent situations (Table 3).

**Table 3:** Patient flow during the COVID-19 pandemic. Significant differences in bold

	Only GPs	Multi-professional	*p*-value
**Please indicate whether the following incidents occurred in this practice since the COVID-19 pandemic:**			
A patient with a fever caused by an infection other than COVID-19 was seen late due to the fact the COVID-19 protocol was followed which delayed the care	33.6%	42.1%	**< 0.001**
A patient with an urgent condition was seen late because he/she did not come to the practice sooner	62.3%	59.9%	0.161
A patient with a serious condition was seen late because he/she did not know how to call on a GP	26.8%	30.7%	**0.018**
A patient with an urgent condition was seen late because the situation was assessed as non-urgent during the telephonic triage	15.3%	22.8%	**< 0.001**
A patient with an urgent condition other than COVID-19 was assessed incorrectly during the triage procedure	26.4%	26.9%	0.104

In “Only GPs” practices, 72.3% of respondents agreed or strongly agreed that their workload had increased during the pandemic, compared to 81.1% in “Multiprofessional” practices (*p* < 0.001). Among those in “Only GPs” practice who experienced an increase in workload, 19.3% reported feeling happy with it, while 25.1% felt unprepared. The corresponding figures for “Multiprofessional” practices were 31.8% (*p* < 0.001) and 22.4% (*p* = 0.070), respectively. In “Only GPs” practices, 29.4% of respondents felt that they required additional training to handle the increased workload, compared to 38.8% in the “Multiprofessional” practices (*p* < 0.001).

### Outreach to vulnerable groups

Table 4 shows that a larger proportion of “Multiprofessional” practices changed their routines towards vulnerable patients during the pandemic, than did “Only GPs”. For instance, a significantly larger proportion of “Multiprofessional” practices reported changing their routines for patients with mental illness, drug abuse, or alcohol abuse. They also reported that to a larger extent, they tried to guarantee the healthcare for patients with chronic diseases and adjusted the healthcare for patients in risk groups for COVID-19. Moreover, a significantly larger proportion of the “Multiprofessional” practices reported that they had made routine measures to protect patients in the waiting room, like placing chairs further apart, providing hand sanitizers, and arranging outdoor waiting areas.

**Table 4:** Outreach to vulnerable groups during the COVID-19 pandemic. Significant differences in bold

	Only GPs	Multi-professional	*p*-value
**In this practice, one or more of the following initiatives were taken since the COVID-19 pandemic:**			
A list was compiled from the electronic medical records for at least one group of patients with a chronic disorder (e.g. all patients taking methotrexate and needing to be seen).	22.1%	33.6%	**< 0.001**
This practice contacted patients with a chronic condition who needed follow-up care.	54.0%	66.4%	**< 0.001**
This practice contacted psychologically vulnerable patients.	29.9%	39.3%	**< 0.001**
This practice contacted patients with previous problems of family violence or with a problematic child-rearing situation.	12.6%	19.1%	**< 0.001**

### Guidelines and recommendations

More practices agreed or strongly agreed that there was sufficient protected time to review new guidelines or relevant and reliable scientific literature, before than during the COVID-19 pandemic. This difference was significant both for “Only GPs” (*p* = 0.035) and “Multiprofessional” practices (*p* < 0.001). However, a greater proportion of “Only GPs” practices agreed or strongly agreed that there was enough time, both before (*p* = 0.001) and during (*p* < 0.001) the pandemic compared to “Multiprofessional” practices.

**Fig. 2:**
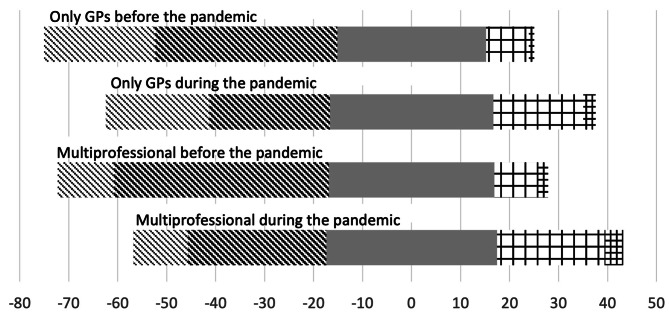
Response to the question “How often is a meeting planned in this practice to discuss existing, new, or amended directives?”. The fields indicate the proportion (%) of the response that are from left to right: Never, Less than once a week, Weekly, Daily, and Multiple times a day

The frequency of meetings to discuss existing, new, or amended directives increased in both types of practices (Fig. 2). The changes from before to during the pandemic were significant for both “Only GPs” (*p* < 0.001) and “Multiprofessional” (*p* < 0.001) practices. Prior to the pandemic, a larger proportion of “Multiprofessional” practices reported having daily or multiple daily meetings, compared to “Only GPs” practices (*p* < 0.001). This trend persisted during the pandemic (*p* < 0.001).

The results of the survey show that a considerable proportion of the responding GPCs, especially in the “Only GPs” group, felt that the guidelines imposed by the government due to the COVID-19 pandemic posed a threat to the good organization of the practice and the personal well-being of the staff. Specifically, among the responding “Only GPs” practices, 34.5% agreed or strongly agreed that the guidelines posed a threat to the good organization of the unit, and 36.5% agreed or strongly agreed that they posed a threat to the personal well-being of the staff in the practice. The corresponding proportions among the “Multiprofessional” practices were 26.5% and 31.9%, respectively. Both differences between the practice types were found to be statistically significant (*p* < 0.001).

## Discussion

The results of this paper demonstrate that “Multiprofessional” practices were more likely to have made structural changes to their reception area, used telephone triage, stopped using the waiting room to protect their patients and staff from infection, and adopted new routines towards vulnerable patients, in other words taking care of safety and quality issues during the Covid-19 pandemic.

These findings are consistent with a recent interview study of team-based practices where structural changes (e.g., waiting rooms) and operational were made to enable safety measures during the pandemic [26].

The finding that “Only GPs” practices were more likely to have performed video consultations and made changes to their daily routines suggests that GPCs with a broader range of healthcare professionals may have more resources and capacity to make structural changes, while GPCs with only GPs may be more agile and adaptable in their day-to-day operations. This finding is consistent with research showing that smaller practices with fewer professionals may have less bureaucracy and a more flexible decision-making process, leading to quicker changes in routines and procedures [6]. This agility may be particularly important during a crisis such as the COVID-19 pandemic when changes in healthcare delivery are needed rapidly. However, an Australian study showed increased use of video consultations during the pandemic in practices with more nurses, but fewer in practices with more allied health professionals (other than nurses) [32]. This points out that the mix of professionals working together is important regarding the use of telemedicine.

Furthermore, professionals in the same facility have more opportunities to interact and share information. Increased interaction might enhance mutual decision-making. Guidelines can be more accepted due to discussions and consensus among colleagues in the team [33]. However, team members in primary care felt disconnected during the pandemic, making communication and coordination difficult [25, 26]. This is interesting and might imply that if teamwork cannot function as usual but turns daily work to more of “everyone doing *their* job” without being able to collaborate as usual with the patients, then perhaps the benefits of teamwork shown in previous studies [8, 33–35] decrease. Maybe teamwork turned more into task-shifting [14] and less collaboration, especially with the increased demands on primary care during the pandemic. Mitzel et al. elaborated on how to preserve the efficiency of teamwork even during difficulties such as the COVID-19 pandemic and in a general situation with staff and resource shortages in parallel to increasing workloads [36, 37] and suggested developing virtual integrated primary care teams [38].

Teaching practices are another issue that enhanced quality and safety organization as well as teamwork.

### Strengths and limitations

The PRICOV-19 study has several strengths that make its findings particularly valuable. First and foremost, it is the largest study ever done in Europe on the quality of care in primary care, with a sample size of over 8,000 primary care GPCs from 38 countries. This extensive sample provides a comprehensive view of the impact of the COVID-19 pandemic on GPCs across Europe. Additionally, the study uses a standardized questionnaire, allowing for reliable and comparable data across different countries and health systems. Furthermore, the study provides insights into the specific changes made by GPCs during the pandemic, which can inform future policies and guidelines to improve the resilience and responsiveness of primary care systems in times of crisis.

One notable limitation of this study is the reliance on a study-specific questionnaire to gather data. Other variables not captured by the questionnaire, may have influenced working routines, guideline adherence, and outreach to vulnerable populations during the COVID-19 pandemic. For example, we cannot tell if the higher use of video consultations among the “Only GPs”-group was to protect against infection or guided by some other reason. Furthermore, the study surveyed participants from multiple countries, each with varying health systems and primary care structures and a large difference in the mix of professions working with GPs within the same practice [6]. As a result, there may be differences in how participants from different countries interpret and respond to the questions, potentially introducing bias or discrepancies in the data. Finally, the analysis did not consider the varying data collection time frames across the participating countries.

## Conclusion

This study indicates that a multiprofessional staff mixture within primary care practices enhances readiness to handle societal challenges like a pandemic, on a structural level, and thereby retain quality and safety for the patients and the staff. Forthcoming policy developments should prioritize promoting such collaboration through multiprofessional teamwork. Additionally, policy initiatives could be implemented to provide support and resources for smaller practices to enhance their capacity to respond to crises such as the COVID-19 pandemic. This could include targeted funding or training programs to assist in adopting new technologies, procedures, and protocols. Finally, policymakers could consider the development of patient-centered care models that balance the benefits of multiprofessional care with the importance of continuity and personalization of care in single practices.

## Data Availability

The anonymized data are held at Ghent University and are available to participating partners for further analysis upon signing an appropriate usage agreement.
